# Editorial: Community series in research efforts, challenges and opportunities in mitigating aflatoxins in food and agricultural crops and its global health impacts, volume II

**DOI:** 10.3389/fmicb.2023.1141308

**Published:** 2023-02-08

**Authors:** Mehdi Razzaghi-Abyaneh, Zhi-Yuan Chen, Mahendra Rai, Masoomeh Shams-Ghahfarokhi

**Affiliations:** ^1^Department of Mycology, Pasteur Institute of Iran, Tehran, Iran; ^2^Department of Plant Pathology and Crop Physiology, Louisiana State University Agricultural Center, Baton Rouge, LA, United States; ^3^Department of Biotechnology, SGB Amravati University, Amravati, Maharashtra, India; ^4^Department of Mycology, Faculty of Medical Sciences, Tarbiat Modares University, Tehran, Iran

**Keywords:** aflatoxins, *Aspergillus*, food and agricultural crops, global health, omics, biocontrol, aflatoxin contamination

Food safety issues have received major consideration not only for increasing numbers of natural and synthetic contaminants but also in relation to ever increasing world population and subsequent demand for food and food supplies. Mycotoxins produced by filamentous fungi are considered as hazardous food and feed contaminants with global distribution. Aflatoxins, the best-known mycotoxins ever, are secondary toxic metabolites produced by diverse groups of filamentous fungi mainly belonging to the *Aspergillus* section *Flavi*. Aflatoxin contamination of food, feed, and agricultural commodities is a serious economic and public health concern not only due to hepatotoxic, teratogenic and carcinogenic effects of this toxin, but also for food spoilage and deterioration result from the growth of producing fungi on susceptible substrates. At present, aflatoxin contamination is a real challenge for developing countries with increasing reports of involvement from new parts of the world due to global warming problem. With expanding our knowledge about detrimental effects of aflatoxins on public health and economy, new technologies are rapidly developed to uncover novel aspects of these dangerous mycotoxins from the taxonomy, biochemistry, and evolution of producing fungi to effective strategies for pre- and post-harvest management of aflatoxin contamination. After a successful endeavor on aflatoxins in volume I (https://www.frontiersin.org/research-topics/9833/research-efforts-challenges-and-opportunities-in-mitigating-aflatoxins-in-food-and-agricultural-crop), this Research Topic as volume II has highlighted recent advances in distribution and management of aflatoxins and aflatoxin-producing fungi in food, food products and agricultural commodities in relation to their impact on public health, economy and agriculture. Within this topic, five articles have been published that complemented our knowledge of the occurrence of aflatoxins, regulatory rules, diversity, and biocontrol of aflatoxin-producing fungi.

In inclusive research on the reduction of aflatoxin contamination in maize, Atehnkeng et al. evaluated the efficacy of “Aflasafe” as a biocontrol product in sub-Saharan Africa, where the agricultural crops especially maize are susceptible to aflatoxin contamination. Aflasafe is composed of a cocktail of atoxigenic *Aspergillus flavus* genotypes native to the region to mitigate aflatoxin contamination. The authors examined Aflasafe in nine maize fields for 3 years, under various conditions to evaluate its biocontrol effectiveness. After quantitation of aflatoxins in grains at harvest and after simulated poor storage, it was shown that biocontrol ability and the genotype viability decreased in a condition which did not support annual treatment. The authors showed that aflatoxin content of maize treated with Aflasafe up to 3 years reduced meaningfully in grains at harvest in comparison with untreated maize. It is interesting to point out that the genotypes were able to survive in the soil in a manner that efficiently reduces maize contamination for 3 years. In conclusion, the authors indicated that although efficient reduction in aflatoxin concentration requires annual applications of Aflasafe, carry-over established in their study can reduce both application frequencies and associated costs.

In a comprehensive review on aflatoxin inhibitors, Ahmad et al. expanded the current knowledge about using natural inhibitors as a sustainable way to combat the spreading of aflatoxigenic fungi and contamination with aflatoxins. Since physical and chemical pre- and post-harvest control strategies of aflatoxin management are laborious and lead to increase in toxic fungicide residues on food and the environment to various extents, the authors discussed various practical and potential application of plants, algae and microorganisms including fungi and bacteria in growth inhibition of aflatoxigenic fungi and subsequent aflatoxin contamination. The role and mode of action of phenolic compounds, antioxidants, plant essential oils, and aromatic compounds such as terpenoids and flavonoids, mushrooms and micro-fungi metabolites especially those obtained from *Lentinula edodes* and *Trametes versicolor*, and finally antimicrobial peptides in inhibiting fungal growth and subsequent aflatoxin contamination was discussed. These natural substances either inhibit the growth of these toxigenic fungi and aflatoxin contamination in food, feeds, and food supplies or suppress aflatoxin biosynthesis and removing or degrading aflatoxins. The authors concluded that extensive studies are still needed to fully comprehend the mode of actions of these natural inhibitors at cellular and molecular levels *via* profiling of transcripts, proteins, and metabolites before they will be applicable for bulk production for industry application.

In a promising study on developing of a chitosan-based natural nanoemulsions as improved antifungal and anti-aflatoxigenic agents, Singh et al. used chitosan nanoemulsion (CsNe) to encapsulate *Aniba rosaeodora* essential oil (AREO-CsNe). Using spectrometry analyses, the authors showed that AREO was successfully encapsulated into CsNe and control released in sufficient amounts. AREO-CsNe was shown to be able to completely inhibit *A. flavus* growth and AFB_1_ more efficiently than that of AREO alone. Regarding the AREO-CsNe mechanism of action, it is believed that the fungal plasma membrane is the main target due to impaired ergosterol biosynthesis and enhancement of fungal cell leakage in AREO-CsNe exposed fungus. *In silico* molecular docking studies also showed efficient interaction of linalool, the main component of *A. rosaeodora* essential oil with Ver-1 and Omt-A proteins, which are known as essential enzymes in AFB_1_ biosynthetic pathway. AREO-CsNe showed enhanced antioxidant activity and in a food model system of stored millets by using *Setaria italica* seeds, induced complete protection against both lipid peroxidation and AFB_1_ contamination in a 1 year period. In conclusion, the authors indicated that enhanced activity of AREO-CsNe over AREO makes it a promising candidate to preserve stored millets and possibly other foods or grains from fungal growth and AFB_1_ contamination.

Contamination of milk and other dairy products with aflatoxin is another major concern and public health problem. Yunus et al. evaluated the importance of knowledge of Farmers and potential ways to control aflatoxin contamination of raw milk in Pakistan. The authors showed that while the majority of the farmers were familiar with the negative impression of fungal-contaminated feed on animal health, <50% of them were conscious of the possibility of transferring aflatoxins from contaminated feed to milk. They concluded that availability of certified aflatoxin-safe feedstuffs, subsidy on quality feeds for animal nutrition, raising Farmer's awareness and legislation and monitoring could effectively impact the fate of aflatoxin contamination of milk.

As a conclusion, data from the present Research Topic highlights the necessity of developing novel strategies for pre- and post-harvest control of aflatoxigenic fungi and aflatoxin contamination of food, feed, and agricultural commodities and expanded our current knowledge about the importance of fungal natural inhibitors and their novel improved formulations in aflatoxin control and prevention strategies.

## Author contributions

All authors listed have made a substantial, direct, and intellectual contribution to the work and approved it for publication.

